# p-Medicine: From data sharing and integration via VPH models to personalized medicine

**DOI:** 10.3332/ecancer.2011.218

**Published:** 2011-08-17

**Authors:** S Rossi, ML Christ-Neumann, S Rüping, FM Buffa, D Wegener, G McVie, PV Coveney, N Graf, M Delorenzi

**Affiliations:** 1 Swiss Institute for Bioinformatics, Lausanne, Switzerland; 2 Fraunhofer IAIS, Schloss Birlinghoven, Sankt Augustin, Germany; 3 Weatherall Institute of Molecular Medicine, University of Oxford, Oxford, UK; 4 Ecancermedicalscience, Gotthardstrasse 20, 6300 Zug, Switzerland; 5 Centre for Computational Science, University College London, London, UK; 6 Department of Pediatric Oncology and Hematology, Saarland University, Homburg, Germany

## Abstract

The Worldwide innovative Networking in personalized cancer medicine (WIN) initiated by the Institute Gustave Roussy (France) and The University of Texas MD Anderson Cancer Center (USA) has dedicated its 3rd symposium (Paris, 6–8 July 2011) to discussion on gateways to increase the efficacy of cancer diagnostics and therapeutics (http://www.winconsortium.org/symposium.html).

Speakers ranged from clinical oncologist to researchers, industrial partners, and tools developers; a famous patient was present: Janelle Hail, a 30-year breast cancer survivor, founder and CEO of the National Breast Cancer Foundation, Inc. (NBCF).

The *p-medicine* consortium found this venue a perfect occasion to present a poster about its activities that are in accordance with the take home message of the symposium.

In this communication, we summarize what we presented with particular attention to the interaction between the symposium’s topic and content and our project.

## The concept

The knowledge about protein coding genes (PCGs), noncoding-RNAs (nc-RNAs), single-nucleotide polymorphisms (SNPs), and their behaviour in the human organism [1–3], which is being obtained from the analysis of data generated by array-based and DNA sequencing technologies, is impressive and is rapidly improving our understanding of the biology of cancer. Moreover, new tools have been developed to predict disease recurrence and progression, response to treatment, as well as new insights into various oncogenic pathways [4–7]. We expect that this will have a strong impact also on treatment decision-making in oncology over the next two decades.

Based on these advances, medicine is undergoing a revolution that is even transforming the nature of health care from reactive to proactive [8] according with the P4 concept that envisages the medicine as a predictive, personalized, preventive, and participatory discipline [9,10], or, in other words, a medicine that will use personal genomic information and other shared data to stratify the diseases in order to give specific treatments and ultimately stop disorder progression.

Expanding the P4 concept, Gorini and Pravettoni [11] indicated a fifth aspect, the psycho-cognitive, in order to give relevance to the behavioural component of the individuals (patients), foster their education and training in interacting with infrastructure like the one *p-medicine* will provide, help them to understand the medical documentation and to finally make well-informed choices (patient empowerment).

The *p-medicine* (www.p-medicine.eu) consortium is creating a biomedical platform to facilitate the translation from current practice to P5 medicine (predictive, personalized, preventive, participatory and psycho-cognitive) by integrating VPH models, clinical practice, imaging and omics data, in order to

- help clinicians in taking therapeutic decisions using models, prediction and prognostic studies
- manage clinical trials
- give the researchers the power to interact with the infrastructure, build reusable workflows and run analyses on several kind of data sources and platforms and enrich the obtained results using previously accumulated knowledge
- provide information for the patients so that they can easily interact with the clinicians and know more about their disease, all in a usable and comprehensible environment.

*p-medicine* works closely together with the Virtual Physiological Human Network of Excellence (VPH–NoE). The VPH–NoE (http://www.vph-noe.eu/) is designed to foster and integrate pan-European research in the field of patient-specific computer models for personalized and predictive health care. Our emphasis is on formulating an open, modular framework of tools and services, including efficient secure sharing and handling of large personalized data sets, performing simulations of disease progression, building tools and models for VPH research [12,13], enrich, evaluate and validate results using literature mining.

## p-Medicine clinical trials

To evaluate and validate the *p-medicine* infrastructure and its usability based on several categories of users (clinicians, patients, bioinformaticians, statisticians, biologists, data managers and tools developers), retrospective and prospective pilot clinico-genomic trials have been selected:

- A Wilms tumour trial will be used to employ the newly developed and validated tools of *p-medicine*.
- Breast cancer trials will be used for the validation of decision-making tools and data acquisition, through sharing, merging and analysing. The breast cancer neoadjuvant pharmacodynamic Bevacizumab phase II trial will be used to extend the VPH tools.
- An acute lymphoblastic leukaemia trial will be used to develop and run a VPH model predicting minimal residual disease and recurrence in childhood acute lymphoblastic leukemia.

## Role of SIB-BCF in *p-medicine*

SIB’s Bioinformatics Core Facility (SIB-BCF) is providing evaluation guidelines and validation scenarios for *p-medicine*, based on its experience in low- and high-level data analysis (normalization, regression, survival analysis, annotation and meta-analysis) heterogeneous data management and experience gained during the ACGT project (http://www.eu-acgt.org/) in the field of quality assurance. SIB-BCF is identifying the objectives that need to be specifically tested (software components and services) in the context of data sources access, patient empowerment tools and clinical trial management.

The evaluation process will be performed in the context of scenarios that can be user group specific, common to several user groups, divided into sub-scenarios.

The *p-medicine* consortium is composed of heterogeneous experts that span from clinicians to bioinformaticians, software developers, data miners and legal attorneys. All together we are defining specific scenarios to be used to test tools of the *p-medicine* infrastructure. SIB-BCF is defining the criteria and the guidelines to support and coordinate the evaluation process. An example of scenario: the dataset GSE22138 (http://www.ncbi.nlm.nih.gov/geo/) was downloaded and further analysed under several aspects.

It contains expression data from uveal melanoma primary tumours, 28 patients that developed metastases and 29 without metastases, with at least 3-year follow-up. After normalization and quality control, differentially expressed genes have been extracted and survival analysis has been performed.

This scenario has at least four different views according with the user’s category:

- Clinician: he/she would like to compare the expected prognosis of a new patient based on the risk index survival curves and check the enriched pathways
- Developer: has the aim to design tools that interface the analysis flow with the p-medicine infrastructure
- Data miner: develop novel methods to analyse all available data, ranging from classical statistical tools over modern bioinformatics methods to novel approaches such as literature mining
- Patient: he/she will visualize the plots, for example the survival ones, with practical explanation, external links that will facilitate the comprehension of the terminology and to enhance the knowledge of the subject as well as the feeling that he/she is crucial part of the decision process.

## Discussion and conclusion

Dr. John Mendelshon, director of the MD Anderson Cancer Center (Prof. De Pinho will become director starting from August 2011) and chairman of WIN listed the main next steps WIN has to take [14]. It is of interest to see that many of these steps are common with those of *p-medicine,* like the following operating procedures:

- Standardization of biobanking and molecular analysis of biopsies
- Audits and compliance standards
- Design of clinical trials, data processing and access to data
- Advertising and fund raising

It is also important to realize that WIN Symposium’s key notes, presentations and discussions shared several main points:

- Patients are willing to participate in trials: “Patients are ready to be engaged, presentation of the available options is the real problem” [15],
- Importance of solve legal aspects to allow the recruitment of patients for a trial and a future reimbursement of biomarker-driven therapies [14,16,17]
- Team work, competition and network of laboratories that share information, methods and data are of utmost importance for going to personalized medicine [16,17],
- Many targets are available: the research community should prioritize them, validate them and transform biomarkers into clinical practice [18,19]
- Bioinformatic approaches are as important as the clinical trial design to make personalized medicine a success story [14]
- Often treatments combining agents work when single agents do not work: Is there a need to approve a combination and not only single agents? [16]
- Selecting the right population to be optimally treated is important [16,18]
- To understand how different treatments and their combination act on the human body, and how resistance and secondary sensitivity can be developed. This includes systematic pathway analysis instead of studying only direct interactions and implies the need for system biology approaches to diseases as the backbone of models [16,17].

Schilsky [15] concluded the Symposium by listing what we still do not know and we should concentrate our efforts on:

- How to understand system perturbations
- How to optimally combine agents (dose, schedule and sequence)
- How to efficiently integrate datasets
- How to quickly convert a laboratory assay to a clinical test
- How to define phenotypes
- How to convey test results to physicians in a useful and intuitive format

As published by MacConaill and Garraway [20], the endless and fast progress in technology and cancer genome biology strongly interacts and fuels the transformation in oncology diagnosis, clinical trial design and treatment. This process needs to be supported by ethical and legal developments. From the organization point of view, clinicians, scientific leaders and all the research community must be ready to face a new phase of education to interpret and use the information that will be produced. Legal issue as well as ways to reimburse the new personalized therapies must be addressed to be ready to turn in reality the many promises developed and that will be discovered to improve patient’s lives.

What emerges from the recent literature and the 3rd WIN symposium is that a minority of leaders are well informed of the issues they have to face accordingly with technological, legal and ethical aspects; the new process of re-organization, training and education will be a major challenge in the near future.

As *p-medicine* consortium, we are glad to be a group of 20 partners that cover all the aspects and backgrounds that are involved in building an infrastructure that will help personalized medicine to become daily clinical practice, as well as helping the subjects involved in train themselves in participating in this new way of treating patients.
